# Stable Levels of Thiol-Oxidised Plasma Albumin, a Biomarker of Oxidative Stress, Is Correlated with Enhanced Performance in Australian Thoroughbred Racehorses

**DOI:** 10.3390/ani15243580

**Published:** 2025-12-12

**Authors:** Christopher James, Jordana Sheahan, Peter Arthur

**Affiliations:** 1 OxiDx Pty Ltd., Nedlands, WA 6009, Australia; 2 Proteomics International, Nedlands, WA 6009, Australia; jordana@proteomics.com.au; 3 School of Molecular Sciences, The University of Western Australia, Crawley, WA 6009, Australia; peter.arthur@uwa.edu.au

**Keywords:** blood biomarker, thoroughbred horse training, thoroughbred horse performance, oxidative stress, performance indicator, muscle recovery, muscle damage

## Abstract

Thoroughbred race performance is influenced by genetics, training, recovery, and management, but decisions about race readiness are often subjective and lack validated physiological markers. This study evaluated thiol-oxidised albumin, a blood-based indicator of oxidative stress, as a potential biomarker of pre-race fitness in Australian Thoroughbreds. Seventy-five clinically healthy racehorses from seven stables were monitored across 216 competitive events (150 races, 66 trials). Blood samples were collected from the jugular at baseline, 48 h pre-race, and for each day up to 8 days post-race to examine levels of thiol-oxidised using the OxiDx test. At baseline, thiol-oxidised albumin levels were stable, but 24% of horses exhibited oxidative stress before racing, rising to 53% after three consecutive races. Thiol-oxidised albumin levels also remained increased for at least 8 days post-race. Statistical analyses were conducted in GraphPad Prism 10.6 using paired t-tests or repeated-measures one-way ANOVA with Dunnett’s post hoc test, alongside a generalised estimating equation model to assess associations with ordinal variables; significance was set at *p* < 0.05. Reference change values were calculated to determine thresholds for biologically meaningful changes in thiol-oxidised albumin levels. Increased pre-race oxidative stress was significantly associated with poorer finishing positions and lower trainer-perceived performance but was unrelated to horse age or sex. Horses without oxidative stress were more likely to finish in the top three. These findings demonstrate that oxidative stress prior to racing is common and adversely affects performance. Thiol-oxidised albumin represents a promising objective biomarker to guide training and racing strategies, with implications for both performance and equine welfare.

## Simple Summary

An inherent part of racing is to elicit a maximal performance from the horse. However, competition or intensive training in horses can damage muscle. Competing with damaged muscle can cause a loss of performance and increases the risk of major injury. Thoroughbred racehorse trainers often rely on their experience and observations to decide if a horse is ready to race, but this can miss hidden signs of stress in the horse. This study used a marker of oxidative stress (the level of thiol-oxidised albumin, OxiDx), which increases when the body has signs of muscle strain and incomplete recovery from hard exercise. We measured this marker in 75 Australian Thoroughbreds before and after their races. We found that one in three horses showed signs of oxidative stress before their very first race, and this increased to over half of horses after three consecutive races. Horses with higher stress levels before a race were more likely to finish further back, run slower, and have their performance rated lower by their trainers. In contrast, horses with lower stress levels were more likely to finish in the top three. These results suggest that many horses may be racing without full recovery, which could affect both performance and welfare. Measuring oxidative stress before competition could help trainers make more informed decisions, reducing the risk of overtraining and injury while improving racing success.

## 1. Introduction

The economic and breeding value of a Thoroughbred racehorse is linked to its success on the racetrack, typically measured by the number of wins achieved during its relatively short competitive career [1]. Race outcomes are influenced by multiple factors, including genetics, competition level, race distance, track surface, environmental conditions, and, critically, the race preparation strategy [2,3]. Training and management decisions regarding a horse’s fitness to compete are largely determined by trainers and owners and often rely on subjective judgement and prior experience rather than objective physiological markers [4].

Race preparation training is designed to establish sufficient cardiorespiratory fitness and musculoskeletal adaptation to reduce the incidence of musculoskeletal injuries (MSI), which remain a key welfare concern in Thoroughbred racehorses [5,6]. Previous research has indicated that both insufficient and excessive volumes of high-speed exercise can elevate MSI risk [6,7,8,9]. Inadequate training exposure results in a poorly adapted musculoskeletal system, whereas excessive workloads may exceed the horse’s recovery capacity, predisposing horses to injury. Notably, Australian Thoroughbreds have been reported to undergo substantially greater monthly training distances at all speeds compared to their UK counterparts. In the UK, monthly workloads exceeding 44,000 m at canter and 6000 m at gallop have been associated with an increased MSI risk, and at least 50% of Australian trainers report workloads surpassing this galloping threshold [10]. These findings suggest that a considerable proportion of Australian Thoroughbreds may be subjected to race preparation training intensities and distances that increase their risk of MSI.

In addition to injury prevention, race-preparation training programmes aim to optimise race day performance through development of musculoskeletal and cardiovascular fitness, as well as neuromotor skills such as balance, coordination, and responsiveness to rider cues [11,12]. Evidence indicates that longer sub-maximal exercise distances are correlated with improved race earnings, suggesting that lower-intensity, higher-distance workloads may be sufficient for achieving race-level fitness [5]. Conversely, prolonged high-intensity workloads may result in overtraining, a syndrome characterised by diminished performance capacity, increased blood lactate concentrations, increased exercise heart rates, reduced appetite, weight loss, behavioural changes, and reluctance to perform strenuous exercise [13,14].

In Australia, Thoroughbred race campaigns typically extend over a minimum of three months, with race starts occurring at intervals of approximately 14–21 days. During this period, horses continue to undergo training between races to maintain fitness. Morrice-West et al. reported that monthly maintenance workloads among Australian training stables were highly variable and influenced by both the intended race distance and the performance class of the horse [5]. Notably, their findings demonstrated that horses galloping less than approximately 4800 m per month achieved higher prize earnings, whereas those exceeding this threshold exhibited a decline followed by a plateau in earnings [5]. These observations parallel those reported for race preparation workloads and suggest that excessive volumes of high-intensity training between race events may negatively affect race-day performance in Thoroughbred horses.

Objective biomarkers capable of assessing the physiological impact of race preparation and maintenance workloads would enhance the capacity to individualise Thoroughbred training programmes, thereby mitigating the risks of injury and overtraining while optimising race-day performance. We have identified thiol-oxidised albumin as a potentially useful biomarker of oxidative stress and muscle damage in both human athletes and Thoroughbred horses [15,16,17,18,19]. Under typical training conditions, thiol-oxidised albumin levels increase following intense exercise but generally return to baseline within 24–48 h [18]. Persistently increased levels indicate sustained oxidative stress and likely muscle damage, signalling inadequate recovery, with a potentially increased risk of injury and reduced physical performance if intense exercise occurs before recovery is complete [15,18,19]. Notably, we have shown that thiol-oxidised albumin levels remain increased for at least seven days in some Australian Thoroughbreds following a single race event [19]. As a consequence, there is potential for sustained oxidative stress in some horses to affect performance in consecutive races.

Given the variability of training regimens for Thoroughbred horses and the evidence that many Australian Thoroughbreds may be subjected to excessive pre-race workloads, this study evaluated whether pre-race oxidative stress was increased. Oxidative stress status was assessed by quantifying the level of thiol-oxidised albumin using the OxiDx test, a sensitive and validated approach for detecting oxidative stress in both human and equine athletes [15,16,17,18,19]. We aimed to determine whether Australian Thoroughbreds were entering races with increased levels of thiol-oxidised albumin and, if so, whether these levels were associated with altered performance in short- to middle-distance races (<2400 m). Thoroughbred horse performance was assessed through an objective measure (finishing position) and a subjective evaluation by the trainer. We hypothesised that horses exhibiting increased pre-race thiol-oxidised albumin would demonstrate reduced performance compared with horses presenting normal levels of this biomarker.

## 2. Materials and Methods

### 2.1. Animals

A power calculation, based on data from a previous study in horses [19], indicated that a minimum of 64 participants was required. To ensure adequate power, 75 healthy Thoroughbred racehorses were recruited from seven racing stables: five in Ascot, Perth, Western Australia, and two in Melbourne, Victoria (Cranbourne and Ballarat). The study cohort comprised 38 geldings, 20 fillies (≤4 years), 14 mares (≥5 years), and 3 colts, aged 2–8 years (mean age 3.5 ± 1.4 years). All procedures were approved by the TeleMedVet Ethics Committee (R323/12, AU-TMV0007) and the University Biosafety Office. Written informed consent was obtained from owners/trainers prior to participation.

All horses were clinically healthy, with no reported injury or illness in the preceding three months, f and trainer confirmation was obtained. Management practices, including feeding, housing, bedding, and daily activity, were recorded and remained consistent across the study period and across stables.

### 2.2. Experimental Design

Each horse underwent blood sampling for baseline, pre-race, and post-race assessments of oxidative stress, measured via thiol-oxidised albumin using the OxiDx test. Baseline samples were collected once daily for six consecutive days following completion of the horse’s pre-race training programme, during which horses were race-fit but had not yet competed in a trial or race, and only low-intensity exercise (walking/trotting) was permitted. While specific quantitative training loads (e.g., distances and speeds) were not collected during the pre-race training period prior to the baseline collection for this study, all horses followed typical Australian Thoroughbred race-preparation patterns involving foundation conditioning and progression to gallop work. Blood was drawn from the jugular vein using a 0.5 mL syringe with a 27 G needle and collected consistently between 07:30 and 08:30 each morning, approximately 1–2 h after daily exercise. Pre-race and post-race samples were obtained using the same procedure. We are not aware of any documented diurnal variation in thiol-oxidised albumin levels. Baseline testing was generally conducted 7–14 days before the first race or trial. For pre-race testing, a single blood sample was collected 48 h prior to each race or trial event and repeated for up to four consecutive events per horse. Pre-race testing was conducted 48 h prior to a race to comply with Racing and Wagering Western Australia regulations; thus, no samples at timepoints closer to the race could be obtained. Consecutive events were defined as those occurring within 21 days and without a rest period (spell) external to the training stable. Following each race, the trainers provided a subjective assessment of each horse’s performance by rating the horse on a scale of 1–10. Trainers were asked to rate the horse’s performance irrespective of its finishing position (i.e., a 1st place finish did not necessarily equate to a 10/10 performance). On the scale, 1 represented very poor performance far below what was expected, 5 represented performances at or near what was expected and 10 represented exceptional performance exceeding what was expected. Post-race testing for oxidative stress was conducted each day for 8 days after the race. During the post-race profiling period, trainers were asked not to modify the maintenance workloads of the horse in any way.

A total of 216 competitive events were included: 150 races and 66 trials. Of these events, 165 were conducted in Perth and 51 in Melbourne. Races were classified as sprints (1000–1600 m; *n* = 188, 87%) or middle-distance (1601–2500 m; *n* = 28, 13%). Trials (800–1200 m) occurred between 09:00 and 13:00, and races (1000–2500 m) between 12:00 and 16:00. All events were held on turf tracks, with race conditions documented. Transport is a potential stressor that could influence oxidative stress and may therefore affect the level of thiol-oxidised albumin. However, the impact of transport was minimised in most horses, as Western Australian horses stabled at Ascot required no transport or were transported only short distances (<10 km) to their race or trial events. A trial is an official pre-race requirement overseen by the Principal Racing Authorities. All horses must complete a satisfactory trial before being permitted to start in an official race. Horses are formally entered, stewards oversee the event, and results are recorded; however, trials do not affect a horse’s official rating, and no prize money is awarded. Although trials do not carry the same financial stakes as official races, horses are still ridden competitively. Performance in trials can influence future race nominations, and the same calibre of jockeys typically ride in both trials and races.

### 2.3. Sample Collection and Analysis

Blood collection for the level of thiol-oxidised albumin analysis was performed by a qualified veterinary nurse at the trainers’ stables. A sampling kit that included an airtight re-sealable bag containing dried blood sample collection devices (Revvity226 Spot Saver RUO Card, product number: GR2261005, Revvity, Melbourne, Australia) stored in the presence of silica gel desiccant was used to collected dried blood samples for the analysis of the level of thiol-oxidised albumin. Prior to sample collection, the veterinary nurse was familiarised with the blood collection protocol. A whole blood volume of 0.5 mL or less was drawn directly into a syringe, free of any anticoagulant, and one drop of the collected whole blood from the syringe was deposited onto the centre circle of each collection device to dry the blood sample and any remaining blood inside the syringe was discarded immediately. The process typically took less than 1 min to complete. The devices were then stored in silica gel desiccant away from sunlight until analysis. Samples were coded to blind laboratory personnel to study group (baseline vs. pre-race). Devices were stored in sealed containers with desiccant, protected from light, until analysis.

The level of thiol-oxidised albumin was quantified using the OxiDx test as previously described [19]. All laboratory analyses were performed by a single researcher with extensive experience in the OxiDx testing methodology and were conducted at The University of Western Australia (Perth, Western Australia). Samples were analysed within 48 h of collection and were stored at ambient temperature during transport to the laboratory (approximately 20–30 °C) and at room temperature (22.5 °C) in the laboratory. Briefly, albumin was extracted from dried blood spots into 20 mM phosphate buffer, bound to Cibacron Blue F3GA agarose, and eluted with 45 mM phosphate buffer (pH 8.2) containing 2.5% SDS.

Capillary electrophoresis was performed on an Agilent 7100 CE system (Agilent, Santa Clara, CA, USA) using bare-fused silica capillaries (50 µm ID, 32 cm length, 24 cm effective length). The capillary was pre-conditioned sequentially with methanol (10 min), double-distilled water (2 min), 0.1 M NaOH (15 min), water (2 min), and background electrolyte (20 min). Separations were run with a 30 mM phosphate buffer (pH 8.2) containing 2.5% SDS at +12 kV, yielding ~71 µA current. Absorbance was recorded at 200 nm. Capillaries were flushed with water (1 min) and electrolyte (3 min) between samples.

Oxidised albumin (OA) was expressed as a percentage of total albumin (OA + reduced albumin, RA) using the formula:

%OA = OA/(RA + OA) × 100

### 2.4. Statistical Analysis

Thiol-oxidised albumin is reported as a ratiometric measure and is expressed as a relative percentage to the level of total albumin. A ratiometric measure provides better precision and accuracy compared to measuring the concentration of thiol-oxidised albumin, as discussed in Lim et al. [16]. A baseline level of thiol-oxidised albumin was calculated for each horse as the mean of their six consecutive daily samples. Changes in the level of thiol-oxidised albumin was expressed as percent change from baseline unless otherwise indicated. Statistical analyses were performed using GraphPad Prism (Version 10.6.0, GraphPad Software, Boston, MA, USA). Group comparisons were conducted using paired *t*-tests or repeated-measures one-way ANOVA with Dunnett’s post hoc test, after confirmation of normality. Reference change values (RCVs) were calculated to determine the threshold for biologically significant changes, incorporating both analytical variation and within-subject variability [20]. Bi-directional RCVs were calculated in Microsoft Excel. Levels of thiol-oxidised albumin exceeding the upper RCV cutoff were interpreted as indicative of oxidative stress [20]. A generalised estimating equation model was used to evaluate association between change in RCV and ordinal variables (placing and perceived performance). Data are presented as mean ± 95% confidence interval (CI), with statistical significance defined as *p* < 0.05.

## 3. Results

Trainers participating in this study reported that both races and trials were perceived as equivalent competitive environments, with horses motivated to perform at their maximal capacity in either setting. Consistent with this perception, no statistically significant difference in performance or effort was detected between the two event types. The mean running speed of horses during official races (16.5 ± 0.5 m/s) was comparable to that observed during trials (16.3 ± 0.4 m/s). Consequently, for the purposes of analysis, trials and races were considered equivalent and are hereafter collectively referred to as “races”.

**Figure 1:**
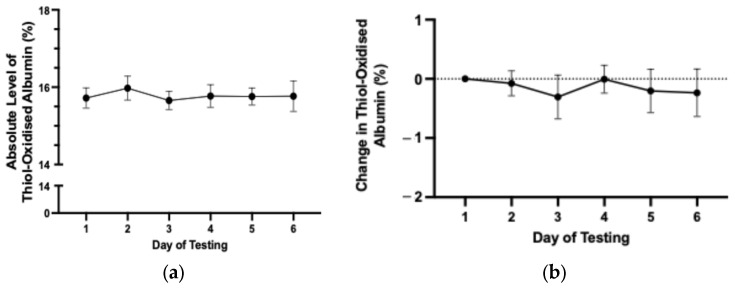
(**a**) The average absolute level of thiol-oxidised albumin for each day of the baseline testing period. (**b**) Change in level of thiol-oxidised albumin from day 1 where thiol-oxidised albumin was set at zero for each horse. Data are means ± 95% CI. *n* = 75 horses.

To assess the day-to-day variability in thiol-oxidised albumin, baseline testing was undertaken 7–14 days before the first race or trial. The average absolute level of thiol-oxidised albumin in race-fit horses over 6 consecutive days did not change significantly and averaged 15.6 ± 0.35% over 6 days of testing (Figure 1a,b).

**Figure 2:**
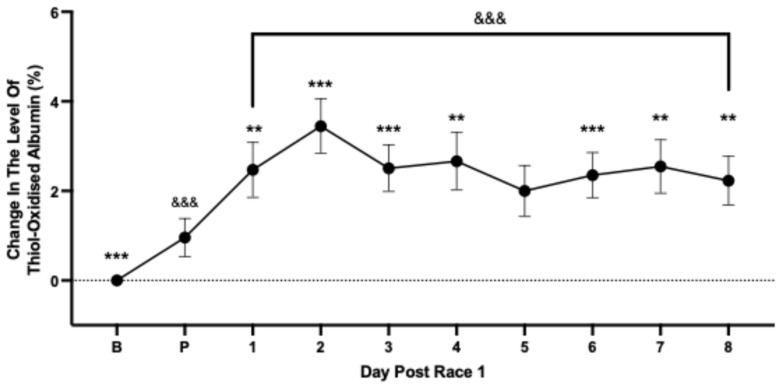
The average change in level of thiol-oxidised albumin from baseline (B, set to 0%) prior to (P) and for each of the days following the first race event. Data are means ± 95% CI. *n* = 65–75 horses. *** significantly different from pre-race (P) *p* < 0.001; ** *p* < 0.01. ^&&&^ significantly different from baseline (B) *p* < 0.001.

To account for between-individual variability in thiol-oxidised albumin, changes in the level of thiol-oxidised albumin were expressed relative to an average baseline value calculated for each race-fit horse (set to 0%) over 6 days. At 48 h prior to the first race event, thiol-oxidised albumin had increased significantly by 0.99% (Figure 2). Following the race, there was a further increase in thiol-oxidised albumin which peaked at 3.45% on Day 2 post-race (Figure 2). The level of thiol-oxidised albumin remained persistently increased and had not returned to baseline levels by day 8 post-race (Figure 2).

**Figure 3:**
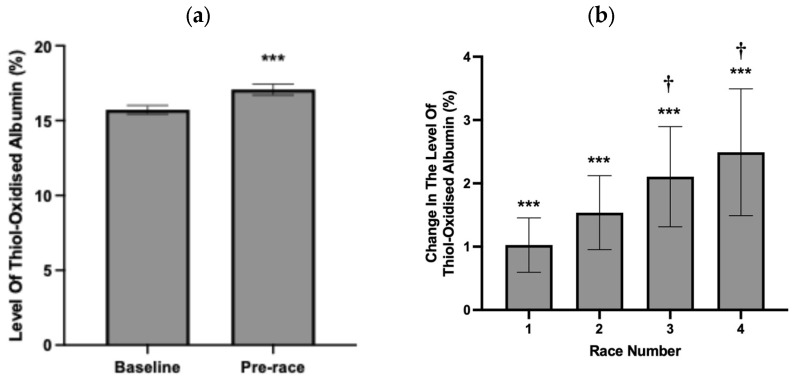
(**a**) Average absolute and change baseline (0%, **b**) in the level of thiol-oxidised albumin at baseline and (**b**) pre-race (48 h prior to race) timepoint for each of the 4 races. Data are expressed as absolute values (**a**) and relative to the pre-race baseline value of 0% (**b**) and are means ± 95% CI. *** significantly different from baseline *p* < 0.001. † Significantly different from race 1 *p* < 0.5. *n* = 75, 60, 35 and 23 horses for baseline, race 1, 2, 3 and 4, respectively.

To assess whether thiol-oxidised albumin was increased prior to consecutive races during a race campaign, the level of thiol-oxidised albumin was measured 48 h pre-race. Across all races, the average level of thiol-oxidised albumin at 48 h pre-race (17.1% ± 0.5 Figure 3a) was significantly higher than the average level of thiol-oxidised albumin at baseline (15.7% ± 0.4%, Figure 3a). Horses participated in up to four consecutive races, and when expressed as change from baseline, the mean change in the level of thiol-oxidised albumin was significantly increased for all races (Figure 3b). Notably, the mean change in the level thiol-oxidised albumin was further increased from pre-race 1 to pre-race 3 and pre-race 4 (Figure 3).

**Figure 4:**
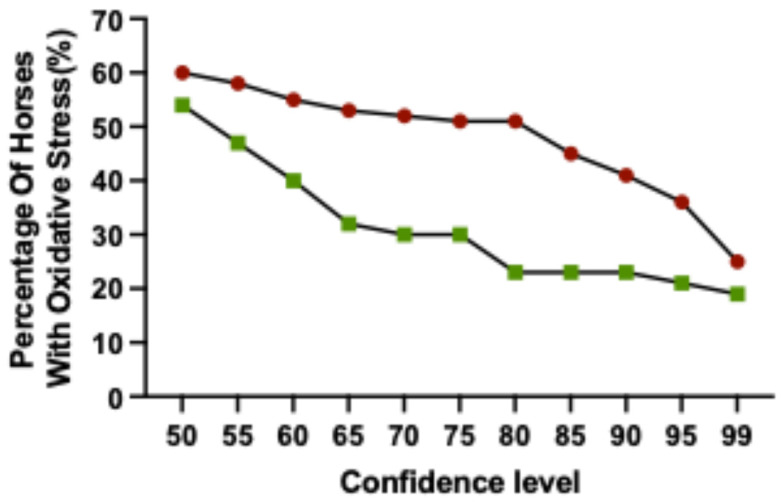
The percentage of Thoroughbred racehorses identified with oxidative stress across varying Reference Change Value confidence levels. Horses were classified as placed (1st–3rd; green) or unplaced (red). *n* = 75 horses.

To identify horses where pre-race levels of thiol-oxidised albumin was considered to be increased, a reference change value (RCV) was calculated for each horse. The RCV was derived from estimates of within-subject variation (CVi) and analytical variation (CVa). Based on six baseline blood samples, the CV total was calculated at 5.7%. The RCV required for an increase to be considered significant was calculated for confidence levels ranging from 55% to 99% (Table 1), with the percentage of racehorses calculated to have oxidative stress at these confidence levels (Figure 4). At low thresholds (e.g., less than 65%) there is an increase in the number of horses that are identified with oxidative stress which could be a result of an increase in false positives. In contrast, at higher thresholds (e.g., 95–99%), fewer horses are identified with oxidative stress and there is a weaker discrimination between horses that placed (1st–3rd) or were unplaced (Figure 4). In the context of practical management of racehorses, there is a distinct difference between placed (1st–3rd) and unplaced horses at 80%, indicating that horses identified with oxidative stress at this level are performance-affected. Trainers participating in this study were consulted regarding acceptable confidence thresholds. After presenting trainers with an explanation of confidence levels and their practical application, all five trainers indicated that 80% was their preferred threshold to identify oxidative stress in their horses. Accordingly, a fractional RCV increase of 1.1074 (fractional increase at 80% confidence) was applied as the cut-off for determining significant increases in the level of thiol-oxidised albumin from baseline values for individual horses (Table 1).

**Figure 5:**
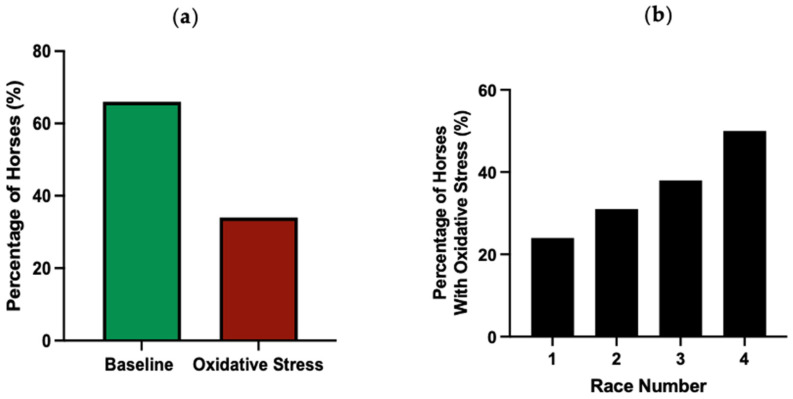
The percentage of horses below (baseline) or above (oxidative stress) their RCV cut-off at 80% confidence prior to all races (**a**) and prior to each consecutive race (**b**). *n* = 75, 60, 35 and 23 horses for race 1, 2, 3 and 4, respectively.

At 48 h pre-race, a substantial proportion of horses exhibited increased thiol-oxidised albumin levels above their individual RCV threshold using a fractional RCV increase of 1.1074 (Figure 5). Across all race events, 34% of horses were classified as racing with oxidative stress (Figure 5a). For consecutive races, there was an increasing percentage of horses with oxidative stress (Figure 5b).

We examined objective and subjective race performance to assess whether the level of thiol-oxidised albumin prior to a race could be affecting horse performance.

The change in the level of thiol-oxidised albumin from RCV for each individual horse was positively correlated with finishing position (Table 2). There was a negative correlation between the change in the level of thiol-oxidised albumin from RCV and the trainers perceived rating of performance (Table 2). There was no correlation between the change in the level of thiol-oxidised albumin from RCV and horse age (*p* = 0.34) or sex (*p* = 0.49). Additionally, there was no correlation between age (*p* = 0.35) or sex (*p* = 0.41) and finishing position. Overall, racing without oxidative stress resulted in a positive likelihood ratio of 1.4 for placing 1st and 1.6 for placing 1st, 2nd or 3rd (Table 3). Racing with oxidative stress resulted in a negative likelihood ratio of 0.24 for both placing 1st and for placing 1st, 2nd or 3rd (Table 3).

**Figure 6:**
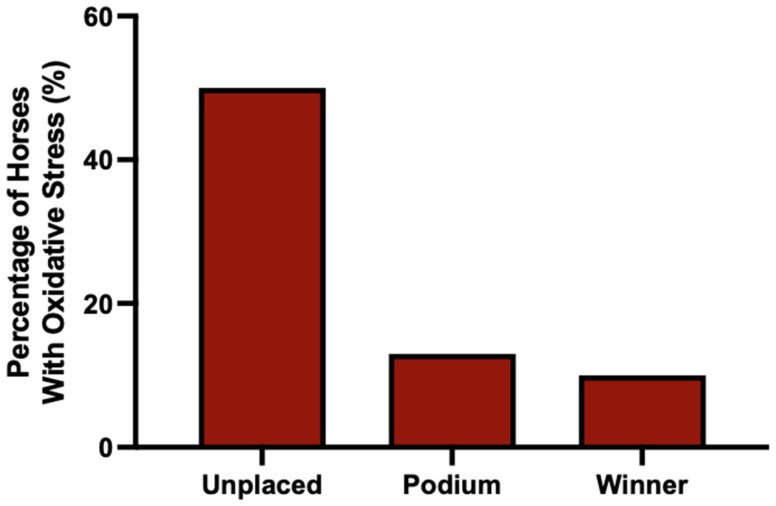
The percentage of horses above their RCV cut-off (evident of oxidative stress) at 80% confidence prior to all races where the horse did not place, finished on the podium (2nd or 3rd), or won the race (1st). *n* = 125, 53 and 37, respectively.

These observations are consistent with results for horses who finished on the podium (i.e., 2nd or 3rd) or who won the race (i.e., 1st). Only 13% of horses who finished on the podium had evidence of oxidative stress, compared to 51% of horses that did not finish on the podium. For first place winners, only 10% of horse had evidence of oxidative stress (Figure 6).

## 4. Discussion

The major finding of this study was that there was evidence of increased oxidative stress, as measured by thiol-oxidised albumin, in a substantial proportion of Australian Thoroughbreds 48 h pre-race, with the proportion increasing with each consecutive race. Notably, lower pre-race thiol-oxidised albumin levels were associated with superior finishing positions and better perceived performance. These results support the concept that increased oxidative stress can limit performance.

To our knowledge, this is the first report quantifying the proportion of Thoroughbred horses with oxidative stress prior to a competitive event. We found that 24% of Australian Thoroughbreds exhibited oxidative stress prior to their first race, with this proportion rising to 53% in horses that undertook three consecutive races. The reason for increased oxidative stress prior to the first race has not been established but may reflect pre-race training practices which were not quantified in this study. The increasing prevalence of oxidative stress through consecutive races may reflect cumulative effects of racing and/or training with insufficient recovery between race events. Further longitudinal investigations are warranted to elucidate the mechanisms underlying these progressive changes in oxidative stress over consecutive race events.

There was a significant and sustained increase in thiol-oxidised albumin levels following a race, which is consistent with our earlier study on Australian Thoroughbred racehorses [19]. Post-exercise increases in thiol-oxidised albumin are related to exercise-induced muscle damage in both humans [15,18,19]. Consistent with these findings, other authors have reported associations between post-exercise oxidative stress and muscle damage in performance horses. For instance, oxidative stress assays such as TBARs and biological antioxidant potential (BAP) activity have been shown to correspond with increased creatine kinase (CK) and aspartate aminotransferase (AST) activities, enzymes that are released following damage to the muscle cell membrane [21,22,23,24,25]. Given that up to 53% of horses in the present study were found to have oxidative stress prior to racing, potentially indicative of muscle damage, these findings indicate a potential welfare issue with increased risk of injury associated with exercise-induced muscle damage.

Our finding that increased pre-race oxidative stress negatively affects Thoroughbred race performance is consistent with results reported in human athlete studies [26,27,28]. We found that race finishing position and trainer-perceived performance were correlated with thiol-oxidised albumin levels, indicating that increased oxidative stress can influence both objective and subjective measures of performance. While we acknowledge that multiple factors, including barrier draw, jockey tactics, and field competitiveness, contribute to race outcomes, our findings suggest that pre-race oxidative stress accounts for a measurable portion of performance variability. Although specific investigations into oxidative stress and equine muscle function (e.g., muscle force output) are limited, evidence from human studies supports the notion that oxidative stress impairs muscle performance [15,27,29,30]. It is therefore plausible that horses racing without oxidative stress are more likely to achieve maximal muscle force output, whereas those with increased oxidative stress experience reduced muscle function, resulting in compromised race performance.

Racing without oxidative stress was associated with an increased likelihood of achieving a top three finish. Positive likelihood ratios of 1.45 (placing first) and 1.7 (placing first, second, or third) indicate an association, suggesting that lower thiol-oxidised albumin levels provide some predictive value for identifying potential winners. In contrast, racing with oxidative stress was associated with a moderately strong negative likelihood ratio for winning and placing. Specifically, a negative likelihood ratio of 0.25 indicates that horses with increased thiol-oxidised albumin levels were only 25% as likely to place first compared to those without oxidative stress, providing evidence that oxidative stress could be a useful predictor for compromised performance.

Several studies have attempted to link parameters (e.g., speed, stride length) from global positioning systems (GPS) to race performance in Thoroughbreds, with mixed results. In a recent study of 485 Australian Thoroughbreds, Schrurs et al. tracked speed and heart rate recovery during and after training sessions [2]. The authors observed a modest relationship between heart rate recovery one minute post high-speed training and finishing speed with race performance; however, the differences were small (~4–6 beats/min; ~1–1.5 s/furlong), limiting practical applicability [2]. In contrast, a similar 2022 study by Schurs et al. found that stride length during training did not predict later racing success in Australian Thoroughbreds [31]. Several other studies in Australian and European Thoroughbreds have reported that total distance covered during training correlated with higher win rates and a greater number of wins and placings per start in the previous season [5,8]. These authors concluded that greater cumulative high-speed work (gallop and race) and racing within the previous 30 days increased the likelihood of winning, while incorporating periods of rest (‘spells’) was associated with higher prize money per start in the preceding season [5,8].

With respect to blood-based biomarkers and physiological measures, several investigations have explored correlations between race performance and parameters such as plasma lactate concentration, heart rate, and uric acid levels, generally finding significant but low correlations, particularly for lactate concentrations [32,33,34,35]. In Korean Thoroughbreds a negative association between red blood cell, haemoglobin concentration and packed cell volume was found for performance rating (subjective values of Korean racing officials), proportion of horses which placed 1st, 2nd or 3rd and prize money per race [36]. However, it should be noted that this analysis was only performed on 21 Thoroughbreds for one race event. Across these studies, results suggest that current GPS-derived physical metrics and biochemical markers provide limited insight into horse performance.

Given the increasing use of GPS in equine monitoring, future studies could investigate how thiol-oxidised albumin levels relate to GPS-derived performance metrics. Our previous work in human athletes demonstrated a relationship between training load and thiol-oxidised albumin, and it would be of interest to determine whether similar associations exist in horses ([37], submitted). Moreover, given our observation that the proportion of horses racing with oxidative stress increased with the number of consecutive races, longitudinal studies following horses through an 8–12-week training and racing programme would provide insight into how thiol-oxidised albumin levels change over time. In addition, as the present cohort consisted solely of Australian Thoroughbreds trained by seven different trainers, further research across international populations is warranted to assess whether differences in training and racing practices influence oxidative stress profiles.

Horse performance is influenced by a wide range of factors, including genetics, training load, health status, race conditions, jockey decisions, and environmental variables (e.g., temperature). As such, evaluating parameters in isolation cannot offer a comprehensive understanding of performance outcomes, which may explain some of the variability seen across studies. While OxiDx testing for the level of thiol-oxidised albumin appears to provide valuable information about oxidative stress and performance, no single analyte can fully account for the complexity of athletic performance during a Thoroughbred horse race.

## 5. Conclusions

Across the equine racing industry, trainers share the objective of developing horses capable of sustained performance throughout their careers. However, race-day preparation remains largely subjective, often guided primarily by prior experience rather than objective measures. Overall, our findings suggest that thiol-oxidised albumin offers a promising tool for evaluating race readiness in Thoroughbreds, particularly given the practicality of the OxiDx testing methodology, which requires only very small blood volumes.

## 6. Patent

The patent applicant for this work is Two Tag Holdings Pty Ltd. The name of the inventor is Peter Arthur. The application number is AU2019240758A1, and the status of the application is awarded.

## Figures and Tables

**Table 1:** RCV fractional increases for confidence levels of 80–99%.

Confidence Level	*p* Value	Z Score	Fractional Increase
50%	0.5	0.674	1.0565
55%	0.45	0.755	1.0633
60%	0.4	0.842	1.0706
65%	0.35	0.935	1.0784
70%	0.3	1.036	1.0869
75%	0.25	1.15	1.0965
80%	0.2	1.28	1.1074
90%	0.1	1.64	1.1380
95%	0.05	1.96	1.1644
99%	0.01	2.58	1.2164

**Table 2:** Correlation between the change in thiol-oxidised albumin from RCV at 80% confidence against variables related to race performance.

Variable	Correlation Coefficient	*p* Value
Finishing position	0.20	0.0001
Perceived rating of performance	−0.16	0.015

**Table 3:** Likelihood ratios for two placing outcomes based on a binary predictor value (thiol-oxidised albumin above or below the RCV).

	Positive Likelihood Ratio	Negative Likelihood Ratio
Outcome 1: Placing 1st vs. not placing 1st	1.49	0.27
Outcome 2: Placing 1st, 2nd or 3rd vs. not placing	1.76	0.26

## Data Availability

The data that support the findings will be available in the UWA research repository at https://research-repository.uwa.edu.au/ following an embargo from the date of publication to allow for commercialisation of research findings. The data supporting the conclusions of this article can be made available by the corresponding author (Chris James), upon request.
